# PET/CT Findings of a Patient with Cardiac Metastasis of Subungual Malign Melanoma

**DOI:** 10.4274/mirt.galenos.2018.59251

**Published:** 2019-09-06

**Authors:** Özgül Ekmekçioğlu, Pelin Arıcan, Şermin Meşe, Nihal Kaplan, Mesut Kafi, Duygu Şimşek, Mehmet Şükrü Ertürk

**Affiliations:** 1 Şişli Hamidiye Etfal Training and Research Hospital, Clinic of Nuclear Medicine, İstanbul, Turkey; 2 Şişli Hamidiye Etfal Training and Research Hospital, Clinic of Medical Oncology, İstanbul, Turkey; 3 Şişli Hamidiye Etfal Training and Research Hospital, Clinic of Radiology, İstanbul, Turkey

**Keywords:** Malign melanoma, PET/CT, subungual, 18F-FDG

## Abstract

A 58-year old patient with a history of subungual malign melanoma was referred to our department for a ^18^F-FDG positron emission tomography (PET)/computed tomography (CT) whole body scan. An unexpected ^18^F-FDG uptake in left ventricule which mimicked either trombus or physiological papillary muscle was detected. Filling defect of intravenous contrast in CT images was also demonstrated in left ventricule cavity. Magnetic resonance imaging scan confirmed cardiac mass with metastatic features of malign melanoma in left ventricule.

## Abstract (tr)

Subungual malign melanom tanılı 58 yaşında erkek hasta tüm vücut ^18^F-FDG pozitron emisyon tomografi (PET)/bilgisayarlı tomografi (BT) görüntülemesi için birimimize başvurdu. PET/BT görüntüleri incelendiğinde kardiyak kesitlerde sol ventrikül düzeyinde fizyolojik papiller kas aktivitesine veya trombüse benzer görünümde artmış ^18^F-FDG tutulumu saptandı. BT kesitlerinde ventriküler kavitede kontrast dolum defekti izlendi. PET/BT sonrasında yapılan manyetik rezonans görüntülemesi ile olguda kardiyak malign melanom metastazı ile uyumlu bulgular saptandı.

## Figures and Tables

**Figure 1:**
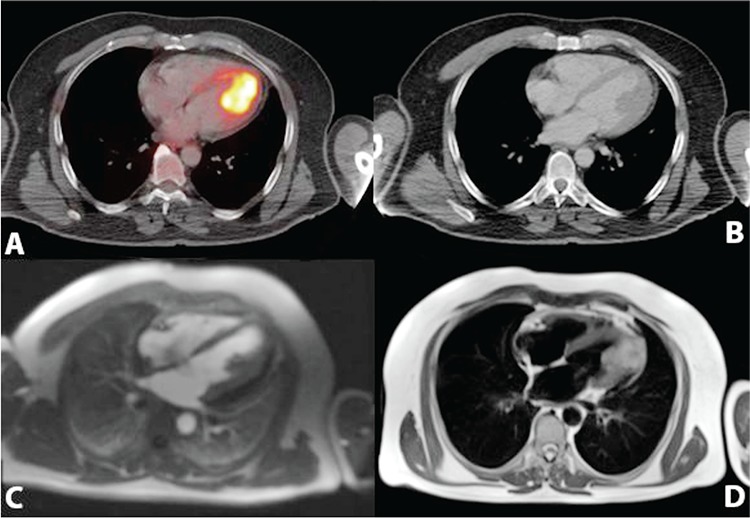
A 58-year old patient with a history of malign melanoma was referred to our PET/CT department for re-staging. Patient was initially diagnosed with excisional biopsy from the nail bed of his first left toe 4 years ago. Inguinal lymph node biopsy revealed negative for metastases at the time of diagnosis. Metastatic lymph nodes were detected in left inguinal region which was confirmed with biopsy 3 years later. Patient was under immunotherapy and had no symptoms either in the control or in the day of scan. PET/CT scan demonstrated increased cardiac (^18^F-FDG) 18-fluoro-deoxy-glucose uptake in left ventricule (A). CT images revealed filling defect of the intravenous contrast in left ventricular cavity which was suggestive of a lesion or a benign pathology like papillary muscle hypertrophy (B). MRI scan showed T1 hyperintensity with gadolinium enhancement in late phase of contrast giving process, 4x3 cm sized T2 hypointensity compatible with melanoma metastasis starting from papillary muscle in apical region, infiltrating through myocardium and extending to pericardium (C, D). Biopsy could not be performed from cardiac mass due to high mortality risk of the patient. Cardiac masses are mostly originated from metastatic spread. Lung cancer, breast cancer and non-hodgkin lymphoma are the most common origins for cardiac metastases (1,2). Malign melanoma has also high potential to metastasize especially to lungs, liver and bones. However, cardiac metastases from melanoma are oftenly detected in autopsy series rather than detected with clinical presentation (3).^18^F-FDG uptake could vary in cardiac tissue and it is usually shown to be helpful in differentiating benign lesions from malignancy (4). In addition to this high uptake in myocardium and the left ventricule can be observed physiologically in ^18^F-FDG PET images (5). Papillary muscle hypertrophy or trombus in ventriculary cavity could show increased ^18^F-FDG uptake in PET images (6,7). As seen in our case, cardiac uptake should be checked carefully to differentiate physiological uptake which could be normally seen in cardiac tissue. Diversely, intravenous contrast is not routinely used in every PET/CT scan protocol. It has been proven to be helpful in identifying pathologic changes in organs with normal findings in unenhanced CT (8). Our images also demonstrated the usage of intravenous contrast with the filling defect seen in left ventricular cavity.
